# Developing Global Health Diplomacy-related Skills Using a COVID-19-like Epidemic Simulation as a Learning Strategy

**DOI:** 10.4269/ajtmh.21-0155

**Published:** 2021-05-10

**Authors:** Miguel Reina Ortiz, Vinita Sharma, Jesse Casanova, Jaime Corvin, Ismael Hoare

**Affiliations:** 1 College of Public Health, University of South Florida, Tampa, Florida;; 2 College of Public Health & Health Professions and College of Medicine, University of Florida, Gainesville, Florida;; 3 International Programs, USF Health, University of South Florida, Tampa, Florida

## Abstract

Public health and global health practitioners need to develop global health diplomacy (GHD) skills to efficiently work within complex global health scenarios, such as the current coronavirus disease (COVID-19) pandemic. Problem-based learning was used as a framework to create a scenario-based activity designed to develop GHD-related skills. The application and effectiveness of this scenario-based activity to develop GHD-related skills were assessed. A mixed-methods approach involving a self-administered survey and one focus group discussion was used. The survey collected baseline participant characteristics as well as understanding and improvements in GHD-related skills using a 5-point Likert scale. The focus group was audio-recorded and thematically analyzed using both inductive and deductive codes. Data integration was achieved by connecting and weaving. Method and investigator triangulation techniques were used. Participants self-reported significantly better postscenario-based activity responses when asked about their understanding of diplomacy, negotiation, communication, and how to address public health emergencies (*P* < 0.01, Wilcoxon signed rank test). Most participants either agreed or strongly agreed that their GHD-related skills improved with participation in the scenario-based activity (diplomacy = 55.6%; negotiation = 66.5%; communication = 72.2%; addressing public health emergencies = 72.1%). Overall, qualitative data were consistent with results obtained using quantitative methods. The scenario-based activity was effective for improving the self-reported understanding of GHD-related skills. The scenario-based activity was also effective for developing the selected GHD-related skills (as self-reported). This scenario-based activity is likely to reduce cognitive load and avoid participant overload, thereby facilitating learning. Further research is required to elucidate its long-term impact on skills development.

## INTRODUCTION

Global health diplomacy (GHD) is a nascent and fast-growing field that sits at the intersection of public health and foreign affairs, bridging these disciplines with other related fields such as management, law, and economics.^1^ Global health diplomacy can also be defined as the “multi-level and multi-actor negotiation processes that shape and manage the global environment for health.”^2^ GHD concerns itself with the global policy environment for health,^1^ which has produced international health-related binding agreements such as the International Health Regulations.^3^ According to the International Health Regulations, the World Health Organization (WHO) has the capacity to declare a public health emergency of international concern when conditions on the ground warrant it.^3^ Only a few instances of a public health emergency of international concern declaration have occurred since the adoption of the 2015 International Health Regulations, including the current coronavirus disease (COVID-19) pandemic.^4,5^ International agreements, such as the International Health Regulations, require the sophisticated work of public health professionals who master diplomacy (i.e., global health diplomacy professionals). In this work, we discuss the use of a COVID-19-like epidemic as a basis for a scenario-based activity to develop GHD-related skills that are linked to current global health competencies.

The Taxonomy of Educational Objectives (commonly known as Bloom’s Taxonomy) is a systematic framework that allows instructors to identify and classify statements of expectation regarding what students should learn as a result of instructional activities.^6^ Analysis, evaluation, and creativity sit at the pinnacle of the revised Bloom’s Taxonomy.^6^ The use of scenarios simulating real-world events can facilitate the achievement of these taxonomic levels by providing an opportunity to connect the knowledge learned in the classroom to the conditions that practicing professionals might face, which is something we refer to as contextual learning. Scenario-based activities could facilitate the development of required skill-based competencies. Leadership, communication, and systems thinking are part of the Council on Education for Public Health (CEPH) Master of Public Health (MPH) Foundational Competencies.^7^ Consistently, public health practitioners are expected to develop and master leadership, negotiation, mediation (management), and communication skills, among others.^7,8^ In particular, global health practitioners need to be prepared to address public health emergencies in an international and multicultural context. Such professionals also need to understand the complex diplomatic processes that lead to international health-related agreements and instruments that have been negotiated to address increasingly complex global health scenarios. Therefore, GHD is an important component of both the knowledge base and skills that a global health professional should develop. We have identified four basic GHD-related skills: diplomacy skills, negotiation skills, communication skills, and the skills to address public health emergencies (DNCEs). Existing avenues for students to achieve these skills as a result of participating in an integrated and contextual activity with an emphasis on GHD-related processes are rather limited.

Problem-based learning, which is a student-centered model incorporating principles of the Adult Learning Theory, has increasingly been used as an educational approach in undergraduate, graduate, and postgraduate continuing education activities in public health and medicine.^9–13^ The problem-based learning model consists of small-group, active learning encounters that emphasize the “know-how,” as opposed to the “know all,” of traditional approaches.^9,11,13^ Scenarios simulating real-world events comprise an active-learning strategy^9^ that could be used as an innovative extension of problem-based methods when training professional global/public health students. Real-world simulations have been used to master professional skills in other fields as well.^13–15^

Using problem-based learning as a framework, we have developed a simulated international health emergency scenario describing the outbreak of a rapidly spreading emerging respiratory viral disease. This scenario-based GHD activity encourages the use of an interdisciplinary perspective to reach a consensus for possible solutions. The aim of this activity is to provide an opportunity for the initial (i.e., it is one of the first opportunities) and early (i.e., during the student’s first semester in the MPH program) development of DNCEs among students in the Global Health Practice concentration of the University of South Florida MPH program. In addition, this scenario is meant to emphasize the roles of global health stakeholders and the need for culturally appropriate, interprofessional collaboration. We examined the practical and theoretical application as well as the effectiveness of scenario-based approaches to support the early and initial development of competencies associated with a professional public health degree with a focus on global health issues.

## METHODS

### Development of the scenario-based global health diplomacy activity.

Grounded in problem-based learning and adult-learning theory,^16^ the scenario-based activity focused on providing work-relevant and practical information, thus empowering students to take charge of their own learning and encouraging active student involvement in the learning process. The scenario-based activity was developed by taking into consideration the following factors: current international public health environment, including current knowledge of and practices for international emerging infectious disease outbreaks; international public health stakeholders and organizations and their mandates/interests; local contextual and cultural aspects; international agreements and regulations; unexpected circumstances that may affect public health responses; transmission mechanisms of infectious diseases and their likelihood to cross international borders; and international movement of people and goods.

The scenario is delivered as part of an interactive classroom learning experience during which students are provided with basic information about a newly reported outbreak of a respiratory infectious disease that is spreading rapidly across communities, cities, nations, and continents; the scenario shares characteristics with the observed initial spread of COVID-19. Before the experience, students are given a list of resources that provide information regarding previous outbreaks, fundamental information regarding different global health stakeholders, their history, and guiding principles/statements such as strategic plans. Selected international stakeholders included in the scenario are traditional public health institutions like the WHO, international nongovernmental organizations like Doctors Without Borders/Médecins Sans Frontières, international development institutions like the World Bank Group, and officers of a selected local Ministry of Health/government. In addition, a two-step cultural brief is provided to guide the roles and interactions with other stakeholders. First, students are referred to Hofstede’s dimensions of national culture.^17,18^ Second, students are provided with culture cards (developed by one of the co-authors) that summarize the salient cultural aspects of selected host countries.

### Study design.

A concurrent mixed-methods approach was used.^19^ The quantitative phase was completed using a self-administered survey, and the qualitative phase included a focus group discussion. Surveys and focus group discussions were completed in November and December 2018. The results of both phases are integrated and reported together here (please refer to the Mixed-methods approaches to data reporting section of this work).

### Study population.

This scenario-based activity was conducted among students enrolled in and attending a concentration-required class within the Global Health Practice program at our institution. Students were voluntarily recruited to participate. Eligibility was determined based on enrollment in the class. Exclusion criteria included having missed participation in the scenario-based activity and unwillingness to participate in the survey or focus group. Students were reassured about the voluntary nature of their participation and the fact that their participation (or lack of participation) had no bearing on their class assessments. A total of 20 students were enrolled in the class.

### Quantitative data collection methods.

A self-administered survey was used to assess the participants’ understanding of different concepts (communication, diplomacy, negotiation, and public health emergency management) and their relevance to global health and public health practice before and after the scenario-based activity. In addition, the self-administered survey evaluated how the skills associated with those same concepts (i.e., communication skills, diplomacy skills, negotiation skills, and skills to address public health emergencies) improved as a result of participation in the scenario-based activity. These questions used a 5-point Likert scale ranging from strongly disagree to strongly agree (1 = strongly disagree; 2 = disagree; 3 = neutral [neither agree nor disagree]; 4 = agree; and 5 = strongly agree). In addition, the self-administered survey included questions to collect important participant characteristics, such as age, sex, and self-reported professional experience.

### Qualitative data collection methods.

Previous research has estimated that one to three focus groups are required to reach saturation.^16^ Because our target population was small (*N* = 20), and because it would be difficult to include all students in voluntary focus groups, only one was conducted.

A total of five randomly selected participants were recruited for the focus groups as follows. A random number was created and assigned to each student on the roster using Microsoft Excel version 16.27. Students were invited to participate in the focus groups using a predetermined order based on the random numbers until the *a priori-*defined sample size (*N* = 5) was fulfilled. Previous research has shown that between four and six individuals can yield the majority of themes in qualitative methods,^20,21^ and the ideal size for the nonmarketing focus group is believed to be between five and eight individuals.^22^ A total of seven students were invited, and two declined participation.

The final instrument had seven open-ended questions that served as a basis for conducting a semi-structured discussion about the characteristics of the scenario-based activity, its uniqueness, participants’ perceptions of the activity, and lessons learned. Questions were probed when necessary. One researcher facilitated the focus group. Sessions were conducted in a private conference room during December 2018. Sessions were audio-recorded and notes were taken.

### Quantitative data analysis.

Survey data were independently entered in duplicate by two different researchers, followed by a data cleaning step. Data were analyzed using JMP Pro 14.0.0. Appropriate descriptive statistics were estimated for each measurement. Prescenario-based and postscenario-based activity differences were compared using paired nonparametric tests (i.e., Wilcoxon signed rank test). Fisher’s exact test was used to compare self-reported postscenario-based activity skill improvement and self-reported professional experience using simplified variables (i.e., Likert scale scores 1–3 were combined into the “does not agree” category and scores 4 and 5 were condensed into the “agree” category; similarly, “a lot” and “some” professional experience categories were combined into the “at least some” category). The significance level for all analyses was defined *a priori* as 0.05.

### Qualitative data analysis.

Focus group recordings were transcribed verbatim, and the transcriptions were verified for accuracy. Transcripts were uploaded and divided into segments using NVIVO 12 Mac. An initial content-driven codebook was prepared using deductive codes based on the focus group guide. Then, inductive codes were created through an iterative process and added to the codebook. Two researchers participated in a thematic analysis that involved discussing emerging themes and addressing discrepancies. When the final codebook was prepared, the transcript was coded and a thematic analysis was performed using NVIVO 12 Mac.

### Mixed-methods approaches to data reporting.

Connecting and weaving approaches to integrate data at the methods and data interpretation/reporting levels, respectively, were used.^23^ This combination of approaches led to the focus group being selected as a sub-sample of the quantitative survey, thereby connecting the two data collection techniques through their sampling frame.^23^ However, the weaving approach allowed us to integrate results by providing a narrative that weaves between the qualitative and quantitative findings.^23^

Method and investigator triangulations were applied to ensure that a better understanding and description of results were provided.^24^ Method triangulation was secured by using multiple data collection techniques (i.e., quantitative survey and qualitative focus group). Investigator triangulation was achieved by comparing notes, insights, and interpretations among investigators during data analysis.

Finally, trustworthiness was established by credibility.^25^ The researchers constantly repeated their understanding of participants’ responses to the participants and provided them with a summary of their answers.

### Ethical considerations.

This study was reviewed by the institutional review board of the University of South Florida and deemed to be “not human subjects research.”

## RESULTS

### Study population characteristics.

Two students did not attend the scenario-based activity; therefore, they were excluded from participation. A total of 18 students participated in the scenario-based activity and completed the survey (100% participation rate). As shown in Table 1, the majority of participants were female (94.4%; *N* = 17), belonged to the young age group (88.9%; *N* = 16), and had some professional experience (55.6%; *N* = 10). Age and sex were excluded from further analyses because of their lack of variability in the sample population.

**Table 1:** Participant characteristics

	n	%
Age group		
Younger (21–30 years)	16	88.9
Older (31–40 years)	2	11.1
		
Sex		
Female	17	94.4
Decline to answer	1	5.6
		
Professional experience		
None	6	33.3
Some	10	55.6
A lot	2	11.1

### General perceptions about the scenario-based activity.

Most participants (88.9%; *N* = 16) either agreed or strongly agreed that the scenario-based activity was conducted in a professional manner; 88.8% (*N* = 15) agreed or strongly agreed that the moderators were effective, and 83.3% (*N* = 15) agreed or strongly agreed that attending the scenario-based activity was worthwhile.

*“… but [in] this one [activity] both [name of one researcher, moderator 1] and [name of another researcher, moderator 2] were like checking and coming and listening to what is going on.”*

*– P1*

Focus group results demonstrated that participants appreciated the space for finding creative solutions and the power to make decisions.

*“I feel like another role play group activities, it was more of a story line where you followed the complete story line but in this one you had your own control over certain actions.”*

*– P4*

Participants also expressed that emphasizing a global perspective was a novelty because most of other previous activities they had experienced were based on local scenarios.

*“for [name of student organization], a student organization, we [did] a hurricane role play activity.”*

*– P2*

*“And I would say that this activity [the scenario-based activity reported in this manuscript] was based globally…”*

*– P3*

### Overall changes in self-reported skill-related Likert scale scores.

**Figure 1.**
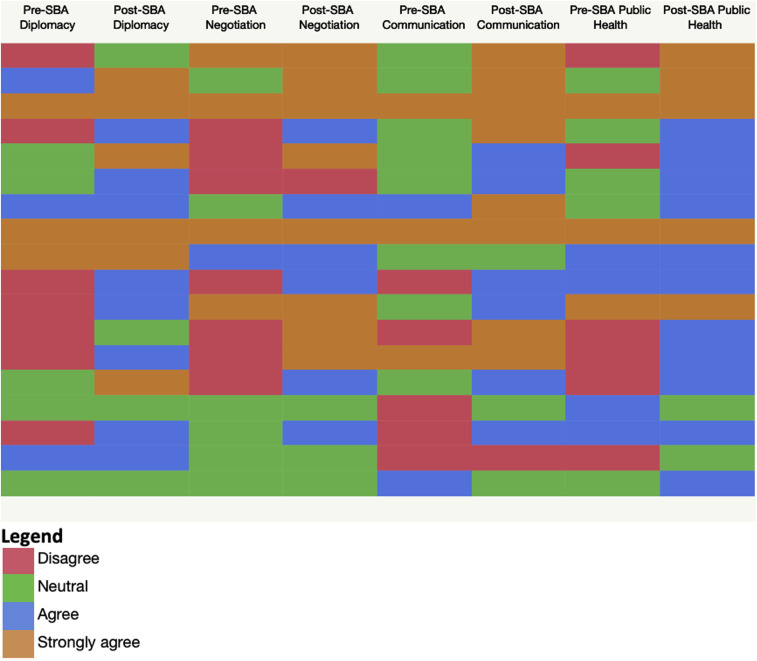
Cell plot matrix of pre-SBA and post-SBA measurements of the self-reported understanding of selected skills using Likert scale scores. SBA = scenario-based activity. For each skill, the preactivity assessment inquired about whether the participants believed that they had a thorough understanding of the skill itself and of how the skill relates to Global Health and Public Health practice. The postactivity statement inquired participants about their perceived improvement in the understanding of the selected skill (and its relevance to Global Health and Public Health practice) as a result of having participated in the SBA. This figure appears in color at www.ajtmh.org.

Figure 1 shows a cell plot matrix representing self-reported prescenario-based and postscenario-based activity Likert scale scores associated with each of the skills studied. Most participants reported a positive migration in the Likert scale between the preactivity and postactivity measurements (Figure 1). Moreover, the majority of strongly agree classifications were observed during the postactivity measurement. Conversely, disagree classifications were confined almost exclusively to the preactivity measurement.

### Impact on the understanding of diplomacy and diplomacy skills.

When asked about whether their understanding of diplomacy was thorough before participating in the activity, only 33.4% (*N* = 6) agreed or strongly agreed, whereas 77.7% (*N* = 14) agreed/strongly agreed that participating in the activity improved such understanding (Table 2). The prescenario-based activity median Likert scale score was 3, whereas the postscenario-based activity median score was 4 (*P* < 0.001, Wilcoxon signed rank test) (Table 3). In addition, a total of 10 (55.6%) agreed/strongly agreed that their diplomacy skills improved as a result of participation in the activity; seven of those participants had at least some level of professional experience (*P* > 0.05, Fisher’s exact test) (Table 4).

**Table 2:** Participants who either agreed or strongly agreed with the preactivity and postactivity statements for each skill studied

	Pre-SBA		Post-SBA	
	n	%	n	%
Diplomacy	6	33.4	14	77.7
				
Negotiation	5	27.8	14	77.7
				
Communication	5	27.8	14	77.7
				
Addressing public health emergencies	7	38.9	16	88.9

SBA = scenario-based activity. Preactivity statement = participants (Likert scale: strongly disagree to strongly agree) indicated that their understanding of the selected skill and its relevance to Global Health and Public Health Practice before participating in the activity was thorough. Postactivity statement = participants (Likert scale: strongly disagree to strongly agree) indicated that their understanding of the selected skill and its relevance to Global Health and Public Health Practice improved as a result of the activity.

**Table 3:** Pre-SBA and post-SBA median Likert scale scores for each skill studied

	Pre-SBA*	Post-SBA*	*P* value (Wilcoxon signed rank test)
Diplomacy	3	4	< 0.001
			
Negotiation	3	4	< 0.01
			
Communication	3	4	0.001
			
Addressing public health emergencies	3	4	< 0.01

SBA = scenario-based activity.

**Table 4:** Self-reported post-SBA selected skill improvement according to the level of professional experience

	Professional experience		
	None	At least some	*P* value (Fisher’s exact test)
	*N* = 6	*N* = 12	
Diplomacy, n (%)			> 0.05
Agree	3 (50.00)	7 (58.33)	
Does not agree	3 (50.00)	5 (41.67)	
			> 0.05
Negotiation, n (%)			
Agree	4 (66.67)	8 (66.67)	
Does not agree	2 (33.33)	4 (33.33)	
			
Communication, n (%)			> 0.05
Agree	4 (66.67)	9 (75.00)	
Does not agree	2 (33.33)	3 (25.00)	
			
Addressing public health emergencies, n (%)			> 0.05
Agree	3 (50.00)	10 (83.33)	
Does not agree	3 (50.00)	2 (16.67)	

SBA = scenario-based activity. Agree corresponds to agree and strongly agree, and does not agree corresponds to neutral, disagree, and strongly disagree on the original Likert scale.

The results of the focus group demonstrated that participants were given opportunities to practice their diplomacy skills during the activity.

*“ ... And we thought [a country] would [deserve our financial investment] because they would be able to mass produce like a vaccine or what...So we were like we’ll fund [that country].”*

*– P4*

*“… but we are taking different perspectives on how we would go ahead and help out the nation”*

*– P5*

### Impact on the understanding of negotiation and negotiation skills.

Before the activity, only 27.7% (*N* = 5) of respondents agreed or strongly agreed that they had a thorough understanding of negotiation, whereas this number increased to 77.7% (*N* = 14) after the scenario-based activity was completed (Table 2). The median Likert scale score before the activity was 3 and the median score after the activity was 4 (*P* < 0.01, Wilcoxon signed rank test) (Table 3). Regarding negotiation skills, 66.5% (*N* = 12) of participants reported that their negotiation skills improved as a result of the activity; this outcome was not affected by the level of professional experience (*P* > 0.05, Fisher’s exact test) (Table 4).

Participants indicated that the activity provided a new approach to negotiation. Participants also expressed their need to develop negotiation skills and recognized that negotiation skills were required (and therefore developed) for the scenario-based activity.

*“…we want to convince [the Minister of Health], I was like…So, how do you convince?”*

*– P1*

### Impact on the understanding of communication and communication skills.

As shown in Table 2, only 27.8% of participants (*N* = 5) agreed or strongly agreed that they had a thorough understanding of communication skills before the activity compared with 77.7% (*N* = 14) after the scenario-based activity (Table 2). The median Likert scale scores were 3 and 4 before and after the scenario-based activity, respectively (*P* = 0.001, Wilcoxon signed rank test) (Table 3). A total of 13 (72.2%) agreed that their communication skills improved after the scenario-based activity, including 75% of those with at least some professional experience compared with only 66.67% of those with none (*P* > 0.05, Fisher’s exact test) (Table 4).

In addition, the findings suggested that participants also learned nonverbal communication skills during the activity. One of the traits mentioned emphatically during the focus group was the appropriate use of body language.

*“So being outside and having to be aware of our body language and how we were acting based on the country really made it [the scenario-based activity] stand out as an activity because otherwise it just would have been…just like everything and every other things that we’ve done.”*

*– P2*

### Addressing public health emergencies.

Only 38.9% (*N* = 7) of students stated before the activity that they had a thorough understanding of how public health emergencies can be addressed compared with 88.9% (*N* = 16) during the assessment after the activity (Table 2). The median Likert scale score increased from 3 before the activity to 4 after the activity (*P* < 0.01, Wilcoxon signed rank test) (Table 3). A total of 13 (72.1%) participants agreed that their skills addressing public health emergencies improved as a result of the scenario-based activity, including 83.33% and 50% of those with some professional experience and no professional experience, respectively (*P* > 0.05, Fisher’s exact test) (Table 4).

This was consistent with the results of the focus group during which participants mentioned confidence in their ability to address international public health emergencies.

*“… as a Minister of Health I could determine the country’s interactions between the other organizations.”*

*– P4*

## DISCUSSION

Global health issues have been recognized as a needed curricular element in medicine and public health by students and faculty alike.^26–28^ Global health issues are inexorably linked to economic development, health inequalities, political stability, security, and peace,^26^ whereas their socioeconomic, environmental, and political determinants are local occurrences that are interconnected on a global scale.^26,28^ Addressing these issues requires intense and complex diplomatic efforts^29,30^; therefore, the development and adoption of international health-related binding agreements, such as the 2005 International Health Regulations, have occurred.^3^ Such agreements and regulations are crucial tools in our fight against global health threats, such as the one posed by the current global COVID-19 pandemic. For instance, the International Health Regulations give the WHO authority to declare a public health emergency of international concern,^3^ a mechanism that was put in practice during the early days of the COVID-19 pandemic.^4^ Major global events, such as COVID-19, may present themselves as cosmopolitan moments,^31^ suggesting the need for a revision of current global health strategies that will require input from experts trained in GHD. Therefore, the development of a skill set for addressing global health issues,^26^ within a diplomatic context, should be an important curricular objective of global health programs.

Scenario-based approaches can be useful during the early development of foundational global health competencies, especially as they relate to diplomacy and negotiation. During this study, problem-based learning was used as a framework to create a scenario-based activity designed to support the early and initial development of DNCEs. The authors and designers of this activity cumulatively had more than 10 years of experience developing and implementing problem-based learning activities, including in international settings such as schools of medicine in Ecuador and the United States, schools of public health (including undergraduate and graduate-level courses) in the United States and Nepal, schools of nursing in Belize, workshops about Geographic Information Systems applied to health in Ecuador, Nepal, and India, and training and development activities with the Peace Corps in Togo, Cameroon, and Senegal, among others. This scenario-based activity places more emphasis on the process rather than on the outcome, thereby reducing the extraneous cognitive load associated with performance anxiety.^32^ By virtue of being a motivating and engaging experience, the scenario-based activity may lead to further reductions in emotional loads.^32^ The scenario-based activity’s briefing and debriefing sessions also reduced the cognitive load^32^ These characteristics are likely to favor learning by helping to avoid surpassing the individual’s working memory capacity (i.e., avoiding overload).^32^

The scenario-based activity also draws from experiential education principles. Specifically, it is an experience that is supported by reflection, critical analysis, and synthesis that encourages students to take initiative and make decisions.^33^ This scenario-based activity may be an effective and efficient method of introducing students to the complex reality of international health regulations and global health actors that allows the acquisition and development of essential knowledge and skills required by global health practice competencies. For instance, participants reported significantly better responses (i.e., either agree or strongly agree) when asked about DNCE knowledge improvement after participation in the activity (*P* < 0.05 for all comparisons, Wilcoxon signed rank test). In addition, most participants either agreed or strongly agreed that their DNCE skills improved after participation in scenario-based activity (diplomacy = 55.6%; communication = 72.2%; negotiation = 66.5%; addressing emergencies = 72.1%). Therefore, the scenario-based activity effectively improved the self-reported understanding of DNCE knowledge and skills. Diplomacy and negotiation, as well as cross-cultural competency (another component incorporated in the scenario-based activity; see Methods section), are among the required global health diplomacy competencies that have been identified by experts and practitioners in the field.^1^

It is noteworthy that this scenario-based activity responds to the needs identified by students, faculty, and national professional and public health-accreditation bodies. For instance, students at our institution had indicated that they would prefer to have applied interdisciplinary team experiences early during their educational program.^34^ The scenario-based activity reported here is delivered during the first semester of the MPH program with a concentration in Global Health Practice. Similarly, our faculty had identified the need to develop an interdisciplinary, experiential, and cross-cutting MPH curriculum.^34^ Alumni and employers had also highlighted the need for the development of systems thinking, professionalism, and communication skills.^34^ In addition, the *Framing the Future: The Second 100 Years of Education for Public Health* report advocates for applied, skills-based, and interdisciplinary programs^34^ that translate to the MPH Foundational Competencies set forth by the CEPH, including leadership, communication, interprofessional practice, and systems thinking.^7^ Accordingly, the University of South Florida’s MPH competencies list, among others, includes *“… effective functioning within and across organizations and as members of interdisciplinary and inter-professional teams.”*^12,34^ The scenario-based activity reported in this work is in alignment with these preidentified needs because it allows students to immerse themselves in the strategic thoughts and actions of different global public health stake holders and represent those actors in an interactive experiential learning opportunity.

In conclusion, the use of a problem-based, learning-informed, systems thinking-oriented, scenario-based global health diplomacy activity is an engaging strategy for the early and initial development of DNCEs in an introductory, required Global Health Practice class at our institution. Such activities should be encouraged and scrutinized for their effectiveness, especially in the long-term. Problem-based learning has been shown to induce long-term learning habits, increase long-term knowledge retention and recall (or prevent knowledge recall decay, at minimum), improve the transfer of concepts to new problems, integrate basic concepts into practical contexts, enhance intrinsic interest in the subject matter, improve confidence, and sustain self-directed learning skills.^13,35^ Importantly, learning about problems in the context in which they will be encountered during professional practice (contextual learning), might facilitate memory coding (at the moment of learning) and recall of knowledge (as a practicing professional).^35^ Some of the concerns raised regarding problem-based learning include increased demand for instructional resources, including teachers, and their corresponding investment and funding needs.^13^ In addition, a critical review of problem-based learning effectiveness suggested that effects on educational outcomes are either small or moderate when they are significant.^11^ Further research is required to assess the mid-term and long-term impacts of the scenario-based activity on self-directed learning habits and knowledge recall and to evaluate the level of resources necessary. Borrowing and modifying a recent view of medical education,^13^ it is important to recognize that high-quality global health education is central to high-quality global health practice.
